# A Case of Congenital Nephrogenic Diabetes Insipidus Caused by Thr108Met Variant of Aquaporin 2

**DOI:** 10.3389/fped.2020.00015

**Published:** 2020-01-30

**Authors:** Lina Ma, Dengyan Wu, Xingmin Wang, Yonghong Yang

**Affiliations:** ^1^Department of Pediatric Nephrology, Lanzhou University Second Hospital, Lanzhou, China; ^2^Department of Nephrology, Gansu Children's Hospital, Lanzhou, China; ^3^Nantong Institute of Genetics and Reproductive Medicine, Nantong Maternity and Child Healthcare Hospital, Nantong University, Nantong, China; ^4^School of Medicine, Jiangsu University, Zhenjiang, China

**Keywords:** congenital nephrogenic diabetes insipidus, aquaporin 2, missense mutation, water reabsorption, autosomal recessive inheritance, T108M variant

## Abstract

Congenital nephrogenic diabetes insipidus (CNDI) is a rare renal disorder caused by mutations in arginine vasopressin receptor 2 (AVPR2) or aquaporin 2 (AQP2). The clinical signs of CNDI include polyuria, compensatory polydipsia, dehydration, electrolyte disorder, and developmental retardation without prompt treatment. In this study we report a rare case of CNDI caused by a single base transition in *AQP2* gene. A 4.5 years old male patient suffered from oral dryness, polydipsia, and polyuria for more than 3 years. Laboratory examinations showed hypernatremia, hyperchloremia, and decreased urine osmolality and specific gravity. Ultrasound and MRI found bilateral upper ureteral dilatation and hydronephrosis. Furthermore, sequencing analysis found a C>T transition leading to a T108M missense mutation of AQP2. The patient was given low sodium diet and treated with hydrochlorothiazide followed by amiloride with indomethacin. The patient's clinical course improved remarkably after 1 year of treatment. This study reports the first case of CNDI featuring T108M missense mutation alone. These findings demonstrate a causative role of T108M mutation for CNDI and contribute to the mechanistic understanding of CNDI disease process.

## Background

Congenital nephrogenic diabetes insipidus (CNDI) is a rare hereditary renal disorder that is characterized by inability of the kidney to concentrate urine in response to antidiuretic hormone arginine vasopressin (AVP), leading to discharge of large volume of unconcentrated urine (1, 2). The clinical signs of CNDI include polyuria, compensatory polydipsia, dehydration, electrolyte disorder (hypernatremia and hyperchloremia), and developmental retardation without prompt treatment (2, 3). The majority of CNDI cases (~90%) are caused by mutations in the arginine vasopressin receptor 2 (AVPR2) gene, leading to an X-linked recessive disorder. Approximately 10% of CNDI cases are caused by mutations in water channel protein aquaporin 2 (AQP2). Of these, 9% of the cases are autosomal recessive inheritance and 1% are autosomal dominant inheritance (2, 4).

AQP2 is a transmembrane protein that is expressed in the principal cells of the kidney collecting ducts and is crucial in maintaining water homeostasis (5). AQP2 is synthesized in the endoplasmic reticulum (ER) and transported to the plasma membrane to form water channels in response to vasopressin (1). *AQP2* gene is located on the chromosome 12q13 and is composed of four exons and three introns encoding the 271 amino acid aquaporin 2. More than 60 CNDI-causing *AQP2* mutations have been identified thus far. Of these, 48 missense mutations, nine small deletions, and one small insertion in the coding sequence and three splicing mutations were identified in CNDI patients (http://www.hgmd.cf.ac.uk/ac/gene.php?gene=AQP2).

In this study we discuss a case of CNDI caused by an AQP2 missense mutation in a 4.5 year old Chinese male. The patient suffered from polyuria, polydipsia, irritability, constipation, and developmental retardation. Laboratory and imaging examinations showed hypernatremia, hyperchloremia, decreased urine specific gravity, and bilateral hydronephrosis. Genetic analysis found a T108M missense mutation in AQP2, confirming CNDI.

## Case Presentation

A 4.5 year old male patient was admitted to the hospital with chief complaint of oral dryness and increased thirst and urinary frequency that had been present for more than 3 years. The patient was an only child, born at 40 weeks by uncomplicated vaginal delivery without significant prenatal complications. He had been breast fed with food supplements as needed. Vaccination was up to date. Apparent growth retardation was noted at admission. The patient developed oral dryness, polyuria, and polydipsia with >3,000 mL liquid intake daily. Notably, the patient's parents are of consanguineous marriage. No other similar cases were reported in the parents' family.

Physical examination at admission showed blood pressure of 95/65 mmHg, respiratory rate of 22 /min, heart rate of 92 /min, and the body temperature of 36.8°C. Body weight and height were 13 kg and 90 cm, respectively, both lower than expected averages of the same age group (18.6 ± 2.3 kg and 109.5 ± 4.4 cm, respectively). No apparent abnormalities in the heart and lungs were noted and physiological reflexes were normal. Gesell Developmental Schedules (6) confirmed developmental retardation as indicated by the Developmental Quotient (DQ): cognitive 86.4, language 74.3, motor 78, fine motor 83.6, and adaptive behavior 80.5, all except cognitive were lower than average.

**Figure 1:**
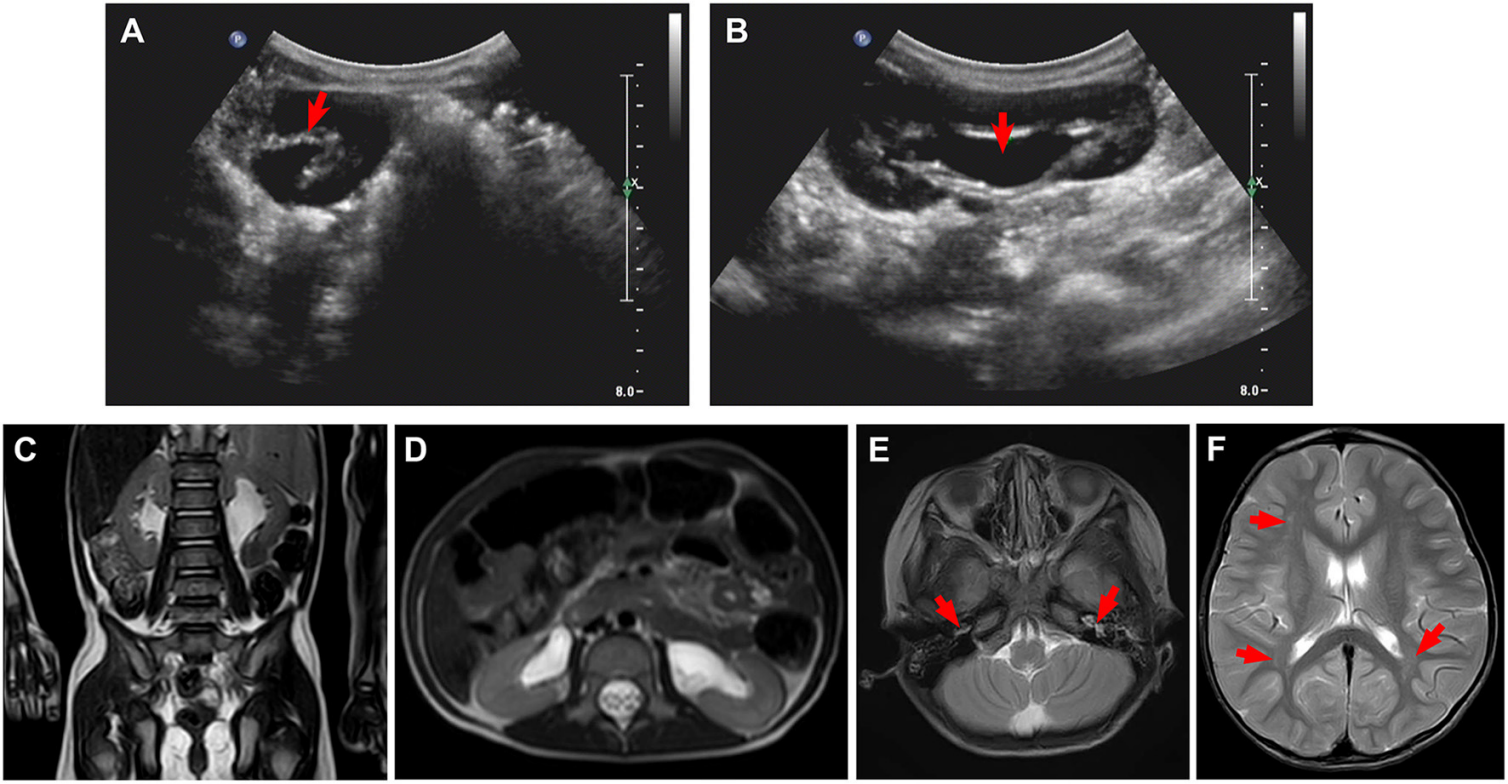
Ultrasound and magnetic resonance imaging (MRI) for kidneys and brain. **(A,B)** Ultrasound shows small crystals in the sinus of both kidneys, bilateral upper ureteral dilatation, and hydronephrosis. **(C,D)** MRI confirms bilateral hydronephrosis and ureteral dilatation, in particular, left side. **(E,F)** Cranial MRI scan shows bilateral mastoiditis (**E**, arrows) and abnormal patchy signal intensity in the anterior and posterior horns of the bilateral ventricles (**F**, arrows), indicating delayed myelination.

Laboratory examinations showed abnormally increased blood sodium and chloride and decreased urine osmolality and specific gravity (Table 1). Ultrasound showed normal sonography of the heart, liver, gallbladder, pancreas, and spleen. Kidney ultrasound, however, showed small crystals in the sinus of both kidneys, bilateral hydronephrosis, and upper ureteral dilatation (Figures 1A,B). No polycystic lesion was seen in either kidney. Magnetic resonance imaging (MRI) confirmed bilateral hydronephrosis and ureteral dilatation, particularly on the left side (Figures 1C,D). Cranial MRI scan showed bilateral mastoiditis (Figure 1E) and abnormal patchy signal intensity in the anterior and posterior horns of the bilateral ventricles, indicating delayed myelination (Figure 1F). To confirm our diagnosis, a water deprivation test was performed for a period of 2 h due to the patient's intolerance to excessive thirst. The results showed increased blood osmolality and unchanged urinary osmolality after water deprivation.

**Table 1:** Changes of laboratory examinations and clinical manifestations after treatment.

	**Before treatment**	**After 1 year treatment**	**Reference ranges**
**Laboratory examinations**			
Specific gravity of urine	1.004↓	1.004↓	1.011–1.025
Blood osmolality (mosm/kg)	317↑	289	280–310
Urine osmolality (mosm/kg H_2_O)	91↓	98↓	550–1100
Blood sodium (mmol/L)	153↑	138	135–147
Blood chloride (mmol/L)	115↑	101	95–110
**Clinical manifestations**			
Volume of liquid intake daily (ml)	>3,000	~1,500	§
Volume of urine daily (ml)	NM	NM	§
Frequency of urination (nighttime)	4–8	≤1	§
Body height (cm)	90	100	§
Body weight (kg)	13	17	§

*↑, Increase compared to reference; **↓**, Decrease compared to reference; NM, not measured; §, varying depends on age*.

**Figure 2:**
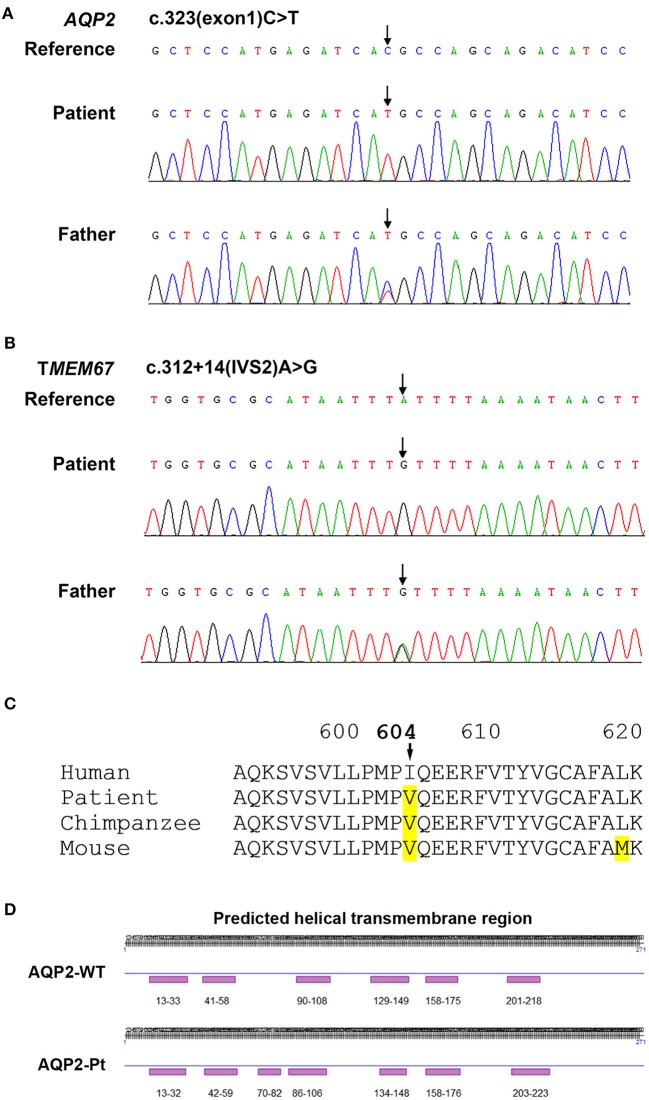
Sequencing analysis for *AQP2* and *TMEM67* genes. **(A)** Sequencing analysis shows a homozygous 323C>T transition in the exon 1 of *AQP2* gene in the CNDI patient (Arrow, *middle*). A heterozygous 323C>T transition occurs at the same position in *AQP2* gene of the patient's father (Arrow, *lower*). **(B)** Sequencing histogram shows a homozygous A>G transversion in the second intron of TMEM67 gene close to the putative splicing site (Arrow, *middle*). The father of the patient harbored a heterozygous mutation at the same position (Arrow, *lower*). **(C)** Sequence alignment of amino acids demonstrates the natural variant of the I604V missense mutation in TMEM67 in chimpanzee and mouse, implying polymorphism. **(D)** Predicted secondary structural changes caused by T108M variation in AQP2.

Based on the clinical picture, laboratory, imaging, and water deprivation test findings, the patient was initially diagnosed with nephrogenic diabetes insipidus (NDI). To determine the potential genetic cause of NDI, exome sequencing was ordered for the patient and his father (genetic test was declined by the patient's mother due to personal reasons). Targets were enriched by NimbleGen Sequence Capture Human Exome 2.1M Array (Roche) and sequenced on HiSeq 2500 System (Illumina). Suspected mutations were confirmed by Sanger sequencing. Genetic test showed that the patient had a homozygous C>T transition at the nucleotide 323 position (c.323C>T) in the exon 1 of *AQP2* gene, leading to a missense mutation at the number 108 amino acid (p.T108M) of aquaporin 2 (Figure 2A). Of note, the father of the patient harbored a heterozygous mutation at the same position in the *AQP2* gene (Figure 2A). Due to the consanguineous marriage of the parents and lack of NDI in patient's mother, she was likely a carrier of the c.323C>T mutation. Taken together with clinical manifestations, patient was diagnosed with congenital nephrogenic diabetes insipidus (CNDI) caused by a missense mutation of AQP2.

## Treatment

To relieve hypernatremia and hyperchloremia, the patient was given a low sodium diet and treated with hydrochlorothiazide 25 mg, twice daily, for 1 month and then switched to indomethacin 12.5 mg, twice daily and compound amiloride hydrochloride (containing amiloride 2.5 mg and hydrochlorothiazide 25 mg per tablet), one tablet daily, to prevent hypokalemia. In addition, alternative therapies including 2 weeks treatment with nerve growth factor (9,000 U, once daily) and 1.5 months treatment with growth hormone (2 IU daily, subcutaneous injection before bed time) were given during the initial therapy. Urine and serum electrolytes were monitored monthly. Patient's clinical course remarkably improved after 2 weeks of treatment and developmental retardation also improved after 1 year of treatment (Table 1).

## Discussion

Under physiological conditions, water balance is regulated by vasopressin-induced signaling pathways that involve water channel protein AQP2 in the apical membrane of principal cells of the renal collecting ducts (2). Binding of vasopressin to type 2 arginine vasopressin receptor (AVPR2) activates stimulatory G protein that, in turn, activates adenylyl cyclase to convert ATP to cyclic AMP. Cyclic AMP, as a second messenger, induces activation of protein kinase A that phosphorylates AQP2 tetramers. Phosphorylated AQP2 tetramers are transported to the apical membrane of principal cells to form water channels for water reabsorption and consequent concentrating of urine.

CNDI is a rare genetic disorder caused by mutations in genes that maintain water homeostasis leading to failure of water reabsorption and urine concentration. Mutations in two genes can cause CNDI. The majority of CNDI is caused by X-linked recessive inheritance of *AVPR2* gene mutations, which accounts for ~90% of all CNDI patients and mainly affects males (4). *AVPR2* gene is located on chromosome Xq28 encoding arginine vasopressin receptor type 2. *AVPR2* gene mutation results in decreased response of principal cells to vasopressin. The remaining 10% of CNDI cases are caused by *AQP2* gene mutations, in which 9% are autosomal recessive inheritance and 1% are autosomal dominant inheritance (4, 7). Autosomal recessive mutations of AQP2 are mainly located within the transmembrane region leading to AQP2 misfolding, incorrect assembly, and ER retention (8, 9), whereas autosomal dominant mutations are located at the C-terminal region that is responsible for AQP2 internalization and trafficking (8, 10). Occasionally, CNDI-causing dominant mutation can also lead to Golgi complex-retention of AQP2 (11). Regardless of recessive or dominant patterns, the consequence of CNDI-causing AQP2 mutations is the defect of water channel on the membrane of principal cells leading to impaired water reabsorption.

To date, more than 60 CNDI-causing AQP2 mutations have been reported. In this study we reported a case of CNDI caused by a single nucleotide substitution (c.323C>T) in *AQP2* gene leading to a missense variant (p.T108M) of AQP2. Notably, this same mutation was observed in a CNDI case that is, however, caused by a small deletion (127-128delCA)-triggered frameshift mutation forming a new stop codon at position 62, leading to the premature termination of AQP2 translation (12). This stop codon occurred prior to the c.323C>T mutation, therefore, whether or not the c.323C>T mutation had a significant role in that CNDI case remained unknown. In contrast, the present CNDI patient is caused by a single c.323C>T transition resulting in the T108M variant, suggesting that the c.323C>T transition is a sufficient causative mutation for CNDI. To assess how this mutation would affect AQP2 function, we performed a *in silico* analysis for AQP2 secondary structure using an online prediction tool (https://predictprotein.org/home). Interestingly, T108M variation may result in the secondary structural changes, in particular, the AQP2 of the patient was predicted to have seven helical transmembrane regions (Figure 2D) instead of six in the wildtype AQP2 (2, 7). Presumably, this change may cause AQP2 misfolding and incorrect assembly. These findings imply that the threonine residue at 108 position is a key site for functional AQP2. Finally, in addition to the c.323C>T mutation in *AQP2* gene, sequencing analysis showed a single nucleotide substitution in the first intron of *AQP2* gene [c.360+3(IVS1)G>A] close to the putative splicing site (Table 2). Whether or not this mutation affects post-transcriptional modification of AQP2, however, is unclear. Further investigation is needed to clarify its potential impact on RNA splicing.

**Table 2:** Mutations detected by Next Generation Sequencing.

**Gene**	**Nucleotide (exon/intron number) variation**	**Amino acid change**	**Homo/Hetero**	**Associated disease**
AQP2	c.323(exon1) C>T	T108M (Missense)	Homo	Autosomal recessive CNDI
AQP2	c.360+3(IVS1) G>A	N/A	Homo	Uncertain
TMEM67	c.312+14(IVS2) A>G	N/A	Homo	Uncertain
TMEM67	c.1066–3(IVS10) C>T	N/A	Homo	Uncertain
TMEM67	c.1810(exon18) A>G	I604V (Missense)	Homo	Uncertain
TMEM67	c.2892(exon27) A>C	T964T (Synonymous)	Homo	N/A

*IVS, intron; Homo, homozygous; Hetero, heterozygous; N/A, not applicable*.

Of note, sequencing analysis found several mutations in the *TMEM67* gene encoding the meckelin, a transmembrane protein that is involved in tissue-specific ciliogenesis and regulation of ciliary membrane composition (Table 2, Figure 2B). Two mutations occurred in exons and the other two mutations occurred in introns of *TMEM67* for this patient. One of these mutations is a homozygous single base transversion (c.2892A>C) in exon 27 of *TMEM67* gene resulting in a synonymous mutation (p.T964T). Another homozygous single base transversion occurred in exon 18 (c.1810A>G) resulting in an I604V missense mutation (Table 2, Figure 2C). TMEM67 mutations result in ciliary dysfunction leading to a broad spectrum of disorders including Meckel syndrome (13), COACH syndrome (14), and nephronophthisis 11 (NPHP11) (15). NPHP11 is an autosomal recessive hereditary disease that mainly affects the tubulointerstitium and has clinical features similar to CNDI, for example, polyuria and polydipsia. However, unlike CNDI, several clinical signs, such as anemia, chronic renal failure, liver fibrosis, and growth retardation are present in NPHP11 patients. In addition, 10–15% of NPHP11 patients have extra-renal manifestations including complications of eyes, brain, and bones, of which retinopathy is the most common feature (15). Laboratory studies for this patient, however, showed normal serum creatinine, blood urea nitrogen (BUN), and hemoglobin, indicating absence of renal failure and anemia. In addition, ultrasound and imaging examinations showed no renal interstitial lesions and extra-renal complications in our patient. Therefore, NPHP11 is less likely to be cause of this patient's presentation. Finally, sequence alignment of amino acids showed that the substitution of isoleucine by a valine at the position 604 in the meckelin existed in other species, such as chimpanzee and mouse (Figure 2C), suggesting that p.I604V variant might have no impact on the structure and function of TMEM67. Further investigations are required to determine whether mutations in the introns (Table 2) affect post-transcriptional modification of TMEM67.

As aforementioned, CNDI patients are incapable of concentrating urine leading to discharge of large volume of unconcentrated urine, which may cause severe dehydration. When long-term, repeated dehydration occurred without proper treatment, ultimately, it could cause crystal deposition in the kidney, nephrolithiasis, and developmental retardation as seen in this patient. Therefore, early diagnosis and intervention are important for preventing CNDI patients from complications, such as damage to urinary and nerve systems and developmental retardation. In combination with clinical signs, imaging examinations and laboratory tests including urine specific gravity, urine osmolality, serum electrolytes, and water deprivation test can help diagnosis of CNDI. In addition, CNDI can also be readily differentiated from other types of diabetes insipidus, such as neurohypophyseal diabetes insipidus via genetic testing (2).

Strategies for CNDI intervention and treatment include restricting sodium intake, supplying with sufficient liquids, correcting hypertonic state induced by hypernatremia and hyperchloremia using thiazide diuretics, and minimizing water discharge using indomethacin or other non-steroidal anti-inflammatory drugs (NSAIDs). In the present case, we gave the patient with a compound amiloride hydrochloride and indomethacin for long-term treatment and short-term alternative therapies with nerve nutrients and growth hormone. Following 1-year treatment, the patient showed remarkably improved symptoms including ameliorated excessive thirst, reduced frequency of urine, particularly during nighttime, and increased body weight and height (Table 1). Medication regimens and nutritional plans used for treating NDI may significantly vary among clinicians. A survey showed that most clinicians (93%) prescribed thiazide for treating NDI, while 62, 55, and 43% clinicians prescribed amiloride, NSAIDs, and indomethacin, respectively (16). Notably, the combination of indomethacin with hydrochlorothiazide is the most common used drug combination for NDI patients, although concerns are raised for gastrointestinal and renal adverse effects of indomethacin (16). In addition, hypotonic fluids, for example 5% dextrose in water, should be used for NDI patients instead of 0.9% saline because it may cause excessive accumulation of sodium chloride and subsequently worsen hypernatremia and hyperchloremia (3). Nonetheless, these therapeutic strategies are unable to fully recover genetic defect-induced poor urinary concentrating mechanism. In addition, these treatments are essential for life, they may cause electrolyte and gastrointestinal disorders as well as negatively impact patient's overall quality of life. Therefore, regular laboratory examination and developmental assessment are needed to evaluate side effects of long-term treatment.

Studies have found several novel therapeutic strategies for AVPR2 mutation-caused CNDI by targeting AVPR2 signaling pathways. For example, chemical chaperones or non-peptide agonists/antagonists can be used to rescue certain endoplasmic reticulum (ER)-retained mutants of plasma membrane protein AVPR2 (17, 18). Although most missense mutations of AQP2 also result in the ER-retention of AQP2, targeted therapies for AQP2 mutation-induced CNDI are less investigated. One study reported that an AQP2 (R254Q) mutant-caused CNDI patients had partial response to 1-desamino-8-D-arginine-vasopressin (dDAVP), leading to improvement of clinical presentation (19). Overall, further investigation into gene therapy is likely to be most efficacious in curing this disease.

In summary, this study presents an autosomal recessive CNDI case that was caused by a C>T transition of *AQP2* gene leading to a missense T108M variant. This is the first CNDI case caused by the T108M mutation alone, suggesting a vital role of threonine 108 for functional AQP2. This study adds new findings to the human gene mutation database. Finally, although developing novel therapeutic strategies for CNDI is important, early diagnosis and intervention are crucial in preventing dehydration-induced damage and developmental retardation, and developing novel therapeutic strategies, such as targeted gene therapies will be an important future pursuit.

## Data Availability Statement

All datasets generated for this study are included in the article/supplementary material.

## Ethics Statement

This study was approved by the Ethical Committee, Lanzhou University Second Hospital. Written informed consent was given by the parents of the child for participation and using clinical records. Written informed consent was obtained from the patient's parents for publication of this case report and all information and any accompanying images contained within it.

## Author Contributions

LM collected the data and prepared manuscript. DW participated in the patient's clinical care and collected the data. XW analyzed the data and wrote the manuscript. YY participated in the patient's care, supervised the study, analyzed the data, and wrote the manuscript.

### Conflict of Interest

The authors declare that the research was conducted in the absence of any commercial or financial relationships that could be construed as a potential conflict of interest.
